# Topics of Public Interest

**Published:** 1910-12

**Authors:** 


### TOPICS OF PUBLIC INTEREST

**Public Provision for Consumptives Doubled.—More than $6,000,000 appropriated.—Still Face Great Lack of Accommodations.**

Sixteen state sanatoria, twenty-eight county hospitals, and twenty-one municipal hospitals for tuberculosis have been erected and provided for since January 1, 1909, says a bulletin of the National Association for the Study and Prevention of Tuberculosis recently issued.

Within the last two years the number of state institutions for tuberculosis has doubled, and the number of county and municipal institutions has increased from about 30 to 80. The expenditures of public money for the treatment of tuberculosis also has more than doubled. Not less than $3,000,000 of state money was appropriated for tuberculosis institutions in 1909, when forty-three legislatures met, and over $600,000 in 1910, when only eleven legislatures were in session. The appropriations of counties and cities for tuberculosis hospitals and sanatoria in the last two years will aggregate fully $2,500,000, bringing the total of official appropriations for tuberculosis hospitals up to over $6,000,000 in the past two years.

In spite, however, of this good showing, the National Association for the Study and Prevention of Tuberculosis states that not one-tenth of the public provision for tuberculosis that is needed has been made. More than 250,000 tuberculosis patients are constantly without proper institutional treatment.

---

### The Census Bureau and Vital Statistics.

### Registration of Infants Dying Under One Year of Age Recommended in New York

AT the first meeting of the Advisory Board recently appointed by Health Commissioner Lederle of New York City to prepare recommendations for the improvement of vital statistics in that city, one of the members of which board is Dr. Cressy L. Wilbur, chief statistician for Vital Statistics of the Bureau of the Census, a recommendation was adopted unanimously to the effect that the most important improvement which could be made in the vital statistics of New York City consists of the verification of the birth registration of every infant dying under one year of age, in order to detect omissions, and strict enforcement of the law providing a penalty for an omission to record a birth in every case thus brought to light.

New York is one of the few cities in the United States having even an approximately complete registration of births, and this action is likely to result in complete registration and so bring its birth statistics up to the standard found in other countries. The Census Bureau is greatly interested in this movement because, as shown in the series of charts prepared for the American Association for Study and Prevention of Infant Mortality, held at Baltimore recently, one of the greatest defects of American vital statistics is the entire lack of reliable rates of infant mortality due to defective birth registration.

---

A CASE OF TETANUS SUCCESSFULLY TREATED WITH MAGNESIUM SULPHATE.—Charles D. Fox, Philadelphia, Pa., gives the history of a case of marked tetanus by means of large injections of tetanus antitoxic serum. The benefit was not pronounced until after the use of an injection of a solution of magnesium sulphate into the spinal canal, when improvement began at once, and went on to a complete recovery.—*Medical Record,* October 22, 1910.

